# Notes on Veterinary Education and Intelligence, Concerning Veterinary and Agricultural Colleges1Read before the Annual Meeting of the U. S. V. M. A., Des Moines, Iowa, September, 1895.

**Published:** 1896-03

**Authors:** W. B. Niles

**Affiliations:** Ames, Iowa


### THE JOURNAL
OF
### COMPARATIVE MEDICINE AND VETERINARY ARCHIVES.

VOL. XVII. *MARCH*, 1896. NO. 3.

### NOTES ON VETERINARY EDUCATION AND INTELLIGENCE, CONCERNING VETERINARY AND AGRICULTURAL COLLEGES.[1]

BY W. B. NILES, D.V.M.,
AMES, IOWA.

THE year just passed has been an eventful one in the history of veterinary education in this country. More changes have occurred in our veterinary colleges than in any previous year; many of which have led to great improvement and will have a far-reaching effect. The action of this Association in raising the requirements for membership is the principal cause of most of these changes. The most earnest advocate of this amendment to our By-laws did not anticipate that its passage would have such a speedy and decided effect upon the advancement of veterinary education. While but three years have elapsed since its passage, all the colleges in this country but three have conformed, or are contemplating conforming, to the requirements of our Association.

In a previous report on veterinary education I divided our colleges into two classes, viz.: State schools—those supported by the State—and private colleges—those incorporated and supported by private parties. In this paper I shall adhere to the same classification.

From the catalogues of the respective colleges and from replies kindly sent in answer to my letters of inquiry I have received the information embodied in these notes.

[1] Read before the Annual Meeting of the U. S. V. M. A., Des Moines, Iowa, September, 1895.

The State colleges having for several years had a course of three years, of about eight months each, have during the year mainly improved by enlarging the course of study and increasing the teaching facilities. As these colleges were well equipped and had long courses of study, the changes have not been so great as in the schools of the other class. The two most important items of interest in connection with this class are the reported closing of the Veterinary Department of the Ohio State University, and the starting of a State veterinary college in New York in connection with Cornell University. It is with much regret that we receive the news of the closing of the veterinary department in Ohio. After several years of hard labor the department had been placed in a position to do good work, work which the State of Ohio can ill afford to lose. Believing that the State schools, with their long course of study, were largely responsible for much of the recent advancement of veterinary education in this country, we also think the profession can ill afford to lose the influence of such a school.

All having the advancement of the profession at heart will be pleased to learn that a veterinary department supported by the State will soon be opened at Cornell University, New York. The editor of the *Review*, our worthy member, Dr. Liautard, said in an editorial notice of this school: "We cannot but feel proud of the step, which will tend to advance the study and teaching of veterinary medicine, which must necessarily be benefited by the prestige of such an excellent university as Cornell." In another place he expressed the opinion that other States will probably within a few years follow the example of New York, and that such action is to be hoped for. I quote as follows from a letter by Dr. Law relative to the curriculum of the proposed department: "While I cannot speak of the curriculum as definitely settled, my own desire is to commence with a four years' course and make it as thorough and comprehensive as circumstances will permit. Quality, rather than quantity, would be my aim, as I feel assured that the country has a right to demand the best results, and that one good man will be of far more value than ten indifferent ones." A department with such a curriculum and aim will surely be an important factor in advancing veterinary education.

The California College, about which many of us have heard, has not that intimate connection with the State university necessary to rank with the State college. In reply to my letter of inquiry, the President of the University of California states that the college is an "affiliated department," having its separate board of trustees and electing its own professors. While we would like to see the school a full-fledged offspring of the State, we are glad to note that it opens as a three-year school, and promises to aid rather than retard the advancement of veterinary medicine.

Many changes may be noted in connection with the private colleges. All of these from which I have received information, with the exception of three (the Chicago Veterinary College, the United States College of Veterinary Surgeons, and the Ontario Veterinary College), have lengthened, or contemplate in the near future lengthening, the course of study to three years. The three excepted have given no intimation of having in view the lengthening of the course of study. It is worthy of note, however, that in two of these some improvements have been made. At the Ontario school it is stated that instruction in the collateral branches is keeping pace with the most recent investigations. This is certainly an improvement, if true. The Chicago college has added the State veterinarian of Illinois to the teaching staff and made some other minor changes. While changes tending to improve the facilities for teaching the collateral branches may have been made, the fact remains that two years of six or seven months each is far too short a time in which to impart a sufficient knowledge of veterinary medicine and surgery, and until these schools lengthen their curricula they will retard rather than advance veterinary education.

Two private colleges, the National, of Washington, and the Ohio, at Cincinnati, advertise to increase the length of the course to three years after the coming session. The Dean of the Indiana Veterinary College writes that "We trust shortly to increase the term of study to three years." The Kansas City College has undergone complete reorganization, and offers a three years' optional course, which it stated will soon be made compulsory. The indications are that this school will take good rank in the near future. The New York College of Veterinary Surgeons and School of Comparative Medicine has undergone many changes for the better during the year. Notable additions to the faculty are Drs. Huidekoper and Hickman; the former occupying the Chair of Anatomy and Surgery, and the latter that of Diseases of Cattle and Obstetrics. The course of study has been lengthened to three years, and graded. With these changes the college should from now on become an important factor in the field of veterinary science.

Regarding changes at the American Veterinary College, which is the first private institution to lengthen the course of study, Dr. Liautard writes "that the facilities for instruction are constantly increasing; that practical work in connection with microscopy, chemistry, etc., is increased more each year; and that the requirements for admission are more severe, this being regulated by a State law recently passed and applying to all veterinary schools in the State of New York."

The McKillip Veterinary College has completed its faculty and in other ways perfected the organization. It is pleasing to note that this school will avoid the great error of some of our private colleges—that is, of allowing anyone to enter, and, almost without exception, all who have entered and remained two years to graduate. The entrance examination is in accordance with that recommended by the Association of Faculties of Veterinary Colleges, and careful examinations are held at the close of each session. It is to be hoped that the example of this and the other private schools that have improved so much may have a wholesome effect on others that have not yet gotten out of the ruts.

In connection with changes in course of study, it should be noted that many of the colleges have recognized the increasing importance of sanitation and demand for sanitarians, and have added instruction in meat- and milk-inspection to the course, and otherwise improved the work in sanitary science.

It seems to the writer that the report of this committee would be incomplete without reference to veterinary education at our agricultural colleges. Of the forty-seven agricultural colleges in the United States twenty-three have a professor of veterinary science; seven others give instruction by employing some outside veterinarian to deliver lectures; and two have a veterinarian on the experiment station staff, who gives no instruction in the college. The other fifteen give no instruction in veterinary science, and have no veterinarian engaged in station work. Some of these intimate that a chair of veterinary science will soon be stablished. Only two of the entire forty-seven have endeavored to establish the work and failed. In those having veterinarians in the faculty, the work consists in giving instruction in the rudiments of the science to agricultural students and such special students as care to take the work. The character of the instruction given is the same in all, only differing in the amount of time devoted to the work. The object in view is to teach the student how to properly care for animals; how to treat some of the more common ailments that must necessarily be looked after by the owner or go without attention, and by the acquirement of this knowledge to learn to understand the proper relations of the educated veterinarian to the live-stock industry. If the student of agriculture can be made to understand that in order to treat intelligently and successfully diseases of our domestic animals, it is necessary to prepare ourselves by long and careful study—in short, that veterinary medicine *is a science*—he will at once distinguish between the educated veterinarian and the empiric. So if the professors of veterinary science only succeed in imparting this single idea they are doing much good. Some may ask, Does the teaching in agricultural colleges tend as indicated, or is the tendency toward empiricism? I unhesitatingly answer that the instruction received does not tend to produce empirics, but, on the contrary, as far as my observation goes, always tends to a higher appreciation of the science, and impresses the student with his incompetency to deal with all classes of cases. I think all who are familiar with veterinary education in agricultural colleges will agree with me in this statement. Such being the results, I attach great importance to the work done by the veterinary professors in our agricultural colleges, and believe it will become a very important factor in veterinary education, particularly in the education of the public to an appreciation of the true relation of veterinary medicine to animal industry. As a sample of what is taught, with the time devoted to the work, I quote from a letter by Dr. McIntosh, of the Illinois Industrial University: "The line of work done in my department are lectures on the anatomy and physiology of the domestic animals during the fall term (three months). In the winter and spring terms (six months) the diseases of farm stock and materia medica are taught. There is a free clinic which supplies us with abundant material on which to study the various ailments." To further illustrate, in Indiana the work is somewhat more extensive, and consists, as I have been informed by the veterinarian in charge at Purdue University, of "anatomy and physiology, with special reference to their value to the stock-raiser." "Stable sanitation, care of breeding animals, and obstetrics are treated quite fully." "Minor surgical operations are taught and the common diseases described." "A free clinic furnishes abundant material for demonstrations." My experience has been that our agricultural college students from these departments make the very best of students for the veterinary college, and the writer believes that the chairs of veterinary science in our agricultural colleges should be in close touch with veterinarians of the country, and receive their hearty support.

State veterinarians and meat-inspectors, in short, all veterinarians holding public positions, are a factor in advancing veterinary education, and deserve mention in this paper. They can assist much in paving the way for the enactment of sanitary laws which will not only affect the health of animals, but will save human lives as well.

Much more might be said, but as my paper is already longer than I intended I will not prolong it further. In closing I wish to kindly thank all who have so willingly assisted me by sending catalogues and letters in answer to my inquiries.

